# Comparison of data fusion strategies for automated prostate lesion detection using mpMRI correlated with whole mount histology

**DOI:** 10.1186/s13014-024-02471-0

**Published:** 2024-07-29

**Authors:** Deepa Darshini Gunashekar, Lars Bielak, Benedict Oerther, Matthias Benndorf, Andrea Nedelcu, Samantha Hickey, Constantinos Zamboglou, Anca-Ligia Grosu, Michael Bock

**Affiliations:** 1 Division of Medical Physics, Department of Diagnostic and Interventional Radiology, University Medical Center Freiburg, Faculty of Medicine, University of Freiburg, Freiburg, Germany; 2 Department of Diagnostic and Interventional Radiology, University Medical Center Freiburg, Faculty of Medicine, University of Freiburg, Freiburg, Germany; 3 German Cancer Consortium (DKTK), Partner Site Freiburg, Freiburg, Germany; 4 German Oncology Center, European University Cyprus, Limassol, Cyprus

**Keywords:** Multiparametric MRI (mpMRI), Prostate specific Antigen (PSA), PSA density (PSAD), Gleason Score, Convolutional neural network, Data fusion, Early Fusion, Intermediate Fusion, Late Fusion, Automated prostate lesion detection, Histological validation

## Abstract

**Background:**

In this work, we compare input level, feature level and decision level data fusion techniques for automatic detection of clinically significant prostate lesions (csPCa).

**Methods:**

Multiple deep learning CNN architectures were developed using the Unet as the baseline. The CNNs use both multiparametric MRI images (T2W, ADC, and High b-value) and quantitative clinical data (prostate specific antigen (PSA), PSA density (PSAD), prostate gland volume & gross tumor volume (GTV)), and only mp-MRI images (*n* = 118), as input. In addition, co-registered ground truth data from whole mount histopathology images (*n* = 22) were used as a test set for evaluation.

**Results:**

The CNNs achieved for early/intermediate / late level fusion a precision of 0.41/0.51/0.61, recall value of 0.18/0.22/0.25, an average precision of 0.13 / 0.19 / 0.27, and F scores of 0.55/0.67/ 0.76. Dice Sorensen Coefficient (DSC) was used to evaluate the influence of combining mpMRI with parametric clinical data for the detection of csPCa. We compared the DSC between the predictions of CNN’s trained with mpMRI and parametric clinical and the CNN’s trained with only mpMRI images as input with the ground truth. We obtained a DSC of data 0.30/0.34/0.36 and 0.26/0.33/0.34 respectively. Additionally, we evaluated the influence of each mpMRI input channel for the task of csPCa detection and obtained a DSC of 0.14 / 0.25 / 0.28.

**Conclusion:**

The results show that the decision level fusion network performs better for the task of prostate lesion detection. Combining mpMRI data with quantitative clinical data does not show significant differences between these networks (*p* = 0.26/0.62/0.85). The results show that CNNs trained with all mpMRI data outperform CNNs with less input channels which is consistent with current clinical protocols where the same input is used for PI-RADS lesion scoring.

**Trial registration:**

The trial was registered retrospectively at the German Register for Clinical Studies (DRKS) under proposal number Nr. 476/14 & 476/19.

## Introduction

In clinical practice, the detection and management of clinically significant prostate cancer (csPCa) often involves a combination of different diagnostic tests and imaging protocols. For diagnosis, staging, and therapy planning, clinicians typically rely on findings from a digital rectal examination (DRE) [1], a prostate- specific antigen (PSA) test and, additionally, the Prostate Imaging Reporting and Data System (PI-RADS) score derived from multiparametric MRI (mpMRI) [2]. To confirm the diagnosis of csPCa patients are required to undergo targeted and systematic biopsy. During this procedure, tissue samples are harvested and analyzed histologically, and a Gleason score is assigned to each sample, from which the lesions can be classified as clinical significant_(Gleason score 7 and above) for further treatment. This parametric clinical data, i.e. PSA [3–6] and Gleason score [7, 8], is combined with image-derived parametric information, such as the prostate volume [8–11], lesion volume, and PSA density [12–16] to retrieve clinically relevant and reliable predictions. However, the multimodal data are not directly correlated among each other [17, 18], due to the different data modalities, and various acquisition techniques. Additionally the variability of the data and the modelling techniques as well as data privacy issues have made it challenging to develop medical multimodal data fusion models [19]. Hence, an analysis of the available image fusion strategies applied to medical imaging and parametric clinical data is urgently needed.

**Fig. 1:**
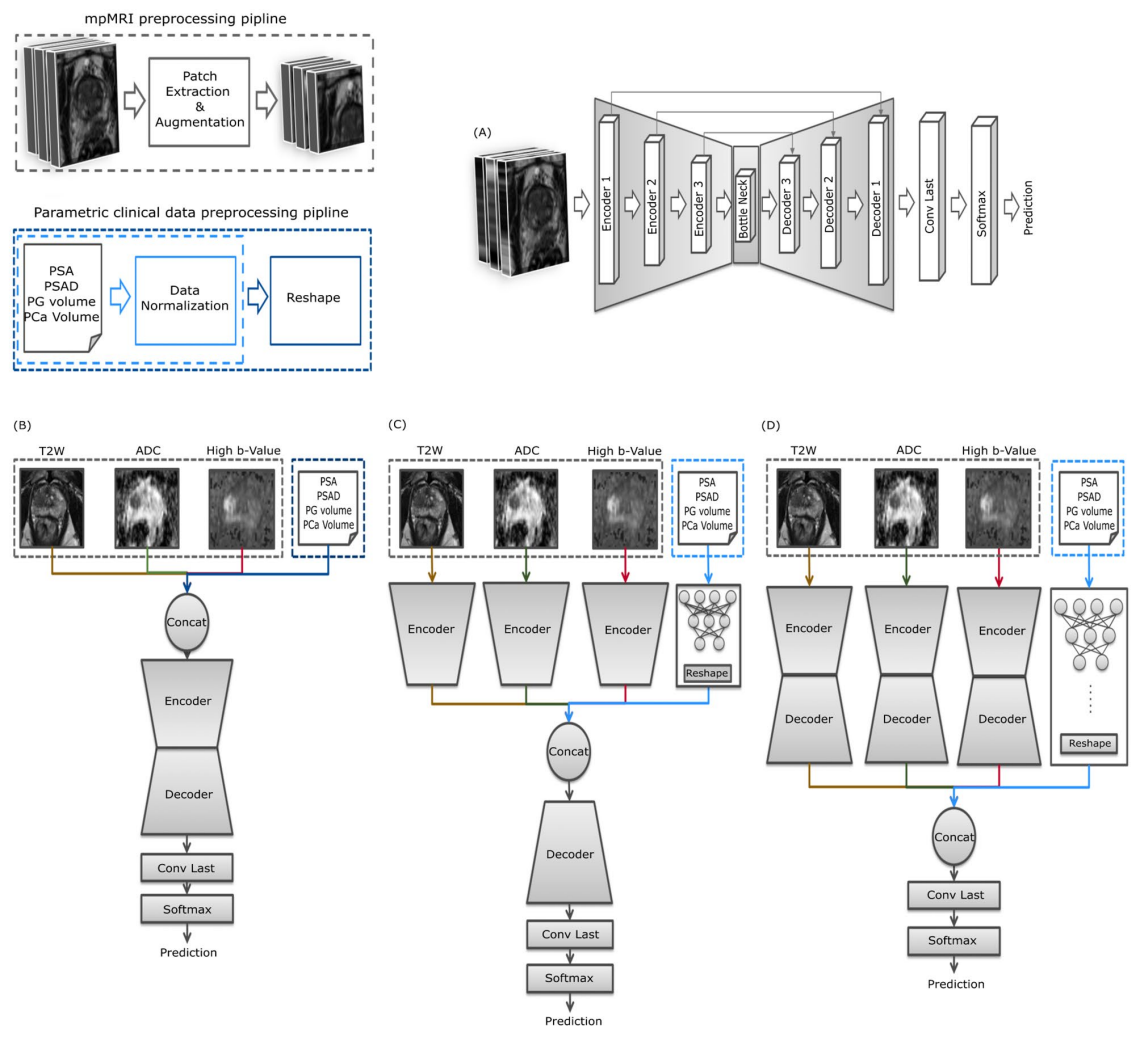
An overview of the mpMRI & parametric clinical data preprocessing pipeline. (**A**) UNet baseline architecture along with an overview of (**B**) the EF, (**C**) IF and (**D**) LF architectures used in this work

Recently, deep-learning-based multimodal data fusion has gained significant interest in the medical community [20], as the combination of diverse data from various modalities can aid the decision process of a convolutional neural network (CNN) [20–22]. A CNN is a type of AI algorithm used to recognize patterns and features in images, such as shapes, colors, edges and textures to make decisions or predictions. It works by passing the image through multiple layers of filters that help identify important details and combine them to understand the overall image. Detailed information on how the CNN process image data can be found in [23–25]. In deep learning, multimodal data fusion refers to fusing data from various imaging modalities, such as magnetic resonance imaging (MRI), computed tomography (CT), positron emission tomography (PET), histology images and non-imaging modalities, such as clinical data from electronic health records, to assist in the decision-making process of CNNs. In the literature, three data fusion techniques have been proposed. (1) In early fusion or input level fusion (EF) (Fig. 1B), the CNN learns a fused feature representation by combining various imaging data and clinical data for decision making. The data from each modality corresponds to one channel in the multi-channel input. (2) In the intermediate fusion or feature level fusion (IF) (Fig. 1C) each channel is passed through multiple CNN layers for feature extraction. These features are then fused and processed further as input to deeper layers of the CNN for decision making. The connection among the early layers enables the CNN to learn the unique feature representation of the corresponding modality, where as the connections from intermediate layers enables the CNN to capture complex relationships between the input modalities, by fully exploiting the feature representation of multimodal images for decision making. (3) In the late fusion method or decision level fusion (LF) (Fig. 1D) the data from each modality is used as input to train independent CNNs. This allows the CNNs to exploit the unique feature representation of the corresponding modality. The outputs of the individual CNNs are then fused and the final prediction is obtained via mean aggregation or by majority voting [26, 27]. Suresh, Harini, et al. [28] used the EF fusion technique to predict onset and weaning of multiple invasive interventions, by integrateing data from all available ICU sources (vitals, labs, notes, demographics), Park, Chihyun and colleagues [29] used EF method for prediction of Alzheimer’s disease based on deep neural network by integrating gene expression and DNA methylation dataset. Peng, Chen, et al. [30] used the EF fusion technique to model features based on the capsule network, to identify breast cancer-related genes. Lee, Garam, et al. [31] used the IF approach to predicting Alzheimer’s disease progression using multi-modal deep learning approach. Huang, Zhi, et al. [32] for survival prediction for breast cancer. Islam, Md Mohaiminul, et al. [33] used the IF stratergy for classification of molecular subtypes of breast cancer. Poirion, Olivier B and coauthors [34] used IF for risk stratification of bladder cancer. Huang and colleagues [35] researched the optimal multi-modal fusion strategy for PCa detection using 2D axial T2w imaging and apparent diffusion coefficient (ADC) imaging as the inputs for their multi-modal fusion model to identify a pipeline that works best for automated diagnosis of csPCa. Reda I, et al. [36] presented a noninvasive CAD system using a meta classifier that integrates PSA screening results in addition to Diffusion weighted MRI based features for prostate cancer diagnosis using the LF technique. Hiremath A, et al [37] implemented a clinical nomogram combining deep learning-based imaging predictions, PI-RADS score, and the clinical data PSA, prostate volume and lesion volume, using multivariate logistic regression to identify csPCa in bi-parametric MRI. They showed the integrated nomogram could help for risk stratification by identifying patients with very low risk, low risk, and intermediate risk of csPCa for active surveillance and very high-risk patients who might benefit from adjuvant therapy using LF.

In this study, we compare three multimodal fusion strategies for automatic clinically significant prostate cancer (csPCa) lesion detection and segmentation. For this, we use mpMRI images (T2W, ADC, high b-value) and the corresponding parametric clinical data (PSA, gross tumor volume, gross prostate volume, and PSA density) as input to train EF, IF and LF networks as shown in Fig. 1(B-D), using an architecture that is based on a 3D-Unet [38]- Fig. 1(A). Specifically, we compare the csPCa detection of a CNN trained with only mpMRI data to a CNN trained with both mpMRI and non-imaging data. Additionally, we compare the lesion segmentation of the network to a commercial deep learning algorithm with EF.

## Materials & methods

### Clinical data

In this study, mpMRI data along with the corresponding parametric clinical data from primary csPCa patients with histologically confirmed cancer lesions was used. The data consists of two groups, with (*n*_*prost*_ = 22) and without (*n*_*irr+prost*_ = 118) whole mount histology data. In the group with whole mount histology data available(*n*_*prost*_), patients initally underwent MRI and during the subsequent therapy the prostate gland was surgically removed (prostatectomy). All other patients underwent radiation therapy.

All MRI studies were carried out between 2008 and 2019 on a clinical 1.5T (Avanto, Aera & Symphony, Siemens, Erlangen, Germany) and 3T (Tim TRIO, Siemens, Erlangen, Germany) MRI systems. The MRI protocol consisted of pre-contrast T2-weighted turbo spin echo (TSE) images in transverse, sagittal, and coronal orientations, diffusion-weighted imaging (DWI) with an echo planar imaging sequence in transverse orientation, and dynamic contrast-enhanced (DCE) MRI images. All the images were acquired with surface phased array (body matrix) coils in combination with integrated spine array coils. The DWI data were acquired with b-values of [0, 100, 400, 800] s/mm^2^ or [0, 250 500, 800] s/mm^2^ for 1.5T, and [50, 400, 800] s/mm² for 3T. From the diffusion-weighted imaging data, a synthetic high b-value image (b = 1400 s/mm^2^) was calculated as recommended by the PI-RADS lexicon [2, 39]. To account for the varying diffusion weightings (b-values) across different field strengths, we generated synthetic DWI images with b = 1400 s/mm², with original b-values for the 1.5T system being [0, 100, 400, 800] s/mm² or [0, 250, 400, 800] s/mm², and for the 3T system, [50, 400, 800] s/mm²] [40]. While no homogenization method was applied to the T2-weighted images due to field strength-dependent tissue T1 and T2 values, we expect similar contrast in the T2-weighted TSE images from both 1.5T and 3T systems, given the comparable T2-values in a wide range of human tissues and the use of repetition times exceeding 5500 ms to minimize T1 contrast [41]. The parametric clinical data consisted of initial PSA values, PSA density, gross prostate gland volume, gross lesion volume. Tables 1 and 2 provide an overview of the parametric clinical data. In addition, clinical scores were derived from the biopsy data such as the Gleason grade, the TNM status, and Gleason grade group [42]. The study was approved by the institutional ethics review board (Proposal Nr.476/14 & 476/19) and patients gave written informed consent.

**Table 1:** Overview of the median along with minimum and maximum age, PSA, PSAD, Prostate gland volume and the prostate lesion volume across the two patient cohorts

Median (Min-Max)	Irradiation & Prostatectomy (*n*_irr+prost_)	Whole mount Histology (*n*_prost_)
**Age**	74 (56–85)	64 (48–76)
**PSA (ng/mL)**	8.8 (2.1–66)	17.4 (6.07–218)
**PSAD(mL or cc**^**3**^)	0.2 (0.1–2.7)	0.47 (0.15–1.67)
**Prostate Volume (mL or cc**^**3**^)	40.8 (10.8–167.7)	42.6 (29.2–130)
**PCa Lesion Volume (mL or cc**^**3**^)	1.8 (0.0–38.1)	11.9 (0.5–76)

Patient data was seperated into a training and a test cohort. The training cohort included a large irradiation and prostatectomy group (n_irr_ + n_prost_ = 118), as training the CNN requires a substantial dataset. Due to the limited number of patients, the test cohort consisted solely of the prostatectomy group (n_prost_ = 22). For the test cohort, post-operative gleason score and tumor volume information were available, along with the ground truth contours from the whole organ histopathology slices, which were co-registered with pre-operative MRI data. The CNNs were trained on T2-weighted images and apparent diffusion coefficient (ADC) maps together with synthetic high b-value images (b = 1400 s/mm²). For all *n*_*irr+prost*_ = 118 and the *n*_*prost*_*=*22 in-house mpMRI data sets, the entire gland (PG-Rad), and the tumor within the prostate (PCa-Rad) were contoured by two experienced radiation oncologists during radiation therapy treatment planning. Images of the whole mount histology slices and the ground truth counters from corresponding whole mount histology slices were acquired as described in [40]. The AI-Rad Companion Prostate MR VA20A_HF02 for biopsy support (Siemens Healthcare AG [43–45]). It performs automated segmentation and automated volume estimation of the prostate and additionally calculates the PSA density, if PSA value is known. The system was used to generate for the csPCa (Rad-AI) contours.

### CNN architecture

As the baseline architecture a patch-based 3D Unet [46] with 3 encoder blocks and 3 decoder blocks (Fig. 1A) was used. Figure 1(B-D) shows the three fusion networks used in this study. For the EF network the input layer consisted of all three mpMRI volumes and parametric clinical data. The parametric clinical data was reshaped to match the dimensions of the image volumes and concatenated with the mpMRI volumes into a single 4D volume. Each channel in the 4D volume corresponds to mpMRI (channel 1 to 3) and parametric clinical data (channel 4 to 7). The IF network consisted of 3 independent encoder heads for each mpMRI volume as input. Each encoder head had 3 encoder blocks, which extracted features from the corresponding mpMRI volumes independently. A shallow multi-layer perceptron (MLP) with three fully connected layers was used to extract features from parametric clinical data. The features from the 3 encoder heads and the MLP head were concatenated at the bottleneck block of the UNet and processed further as input to the decoder blocks in Fig. 1B. For the decision level fusion methods, 3 UNets were trained separately for each mpMRI volume (see Fig. 1C), and an MLP with parametric clinical data as input was trained individually. The final prediction was obtained by mean aggregation of the predicted individual probability scores of these networks as depicted in Fig. 1D. All CNNs were implemented in MATLAB® (2022b, Math Works, Inc., Natick/MA).

### Data preprocessing

To reduce the computation time, the mpMRI data were cropped to a smaller FOV around the prostate. A chance of 70% for a random 2D-rotation (0-360°) in the axial plane was added for data augmentation. Due to the large sizes of the image volumes which would result in system memory issues, calculations were performed on patches of size 64 × 64 × 16 that were randomly chosen with respect to the center location of the original image. Based on the type of fusion network these data were reshaped to match the image dimensions and concatenated along the 4th dimension.

#### Training & testing

All CNNs were trained for 50 epochs on an NVIDIA RTX2080 GPU with a learning rate of 1e-5, a batch size of 4, patch size of 64 × 64 × 16, 50 patches per image, and using the Adam optimizer (Bayesian optimization). The mpMRI data from the prostatectomy group (${n}_{test}$= 22) were used during the testing phase, as the histologic information could be used as a ground truth.

### Performance evaluation

The performance of each network over different epochs was evaluated using precision recall, average precision (AP), F-Score, and Dice Similarity coefficient (DSC). A t-test was performed to identify differences in the predicted scores for these networks.

## Results

**Fig. 2:**
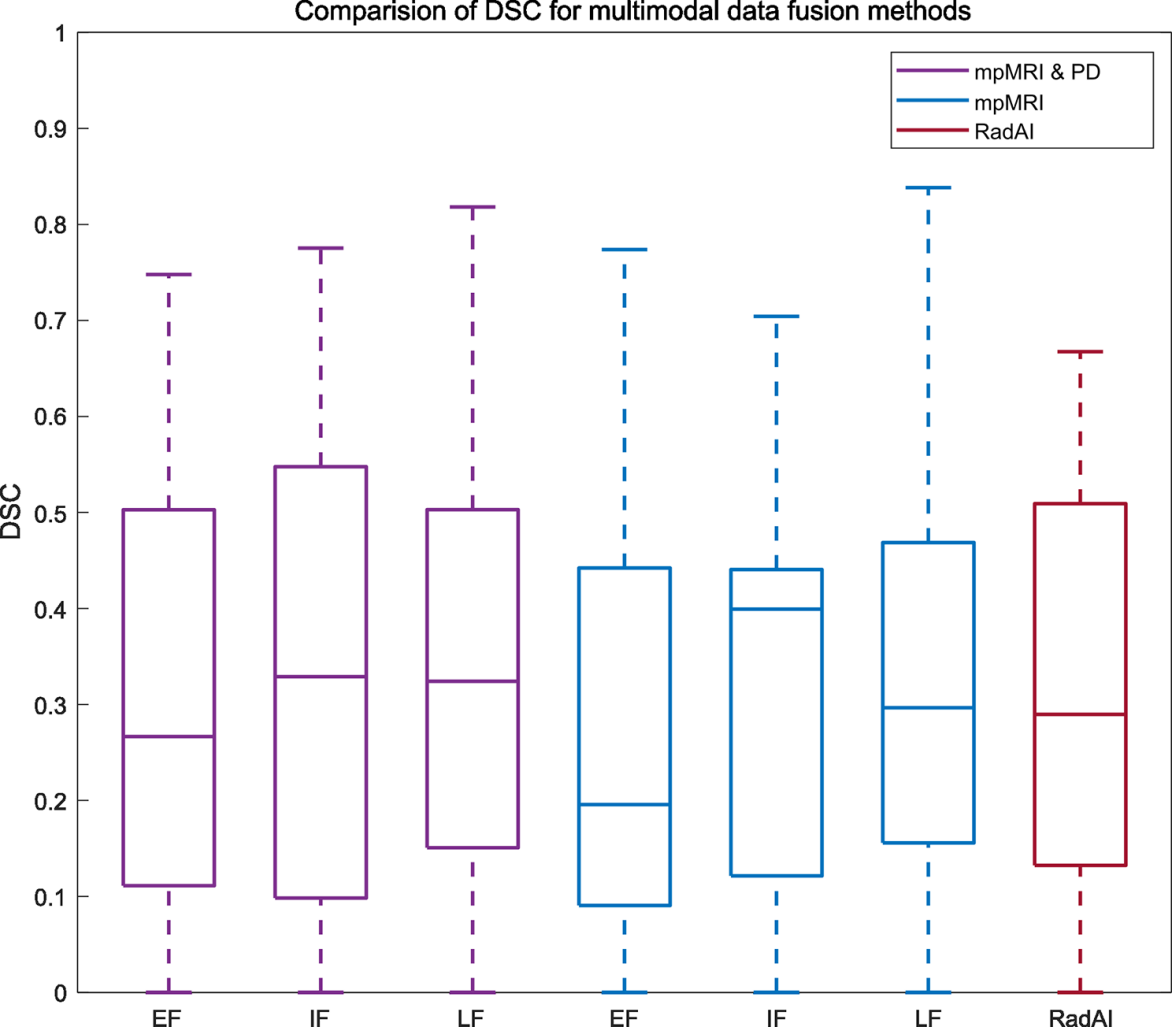
Comparision of DSC for the prediction of csPCa lesion on the test cohort (*n* = 22) for input level, feature level, and decision level fusion networks using mpMRI & parametric clinical data in purple, mpMRI data only in blue and with RADAI in red with the ground truth

**Fig. 3:**
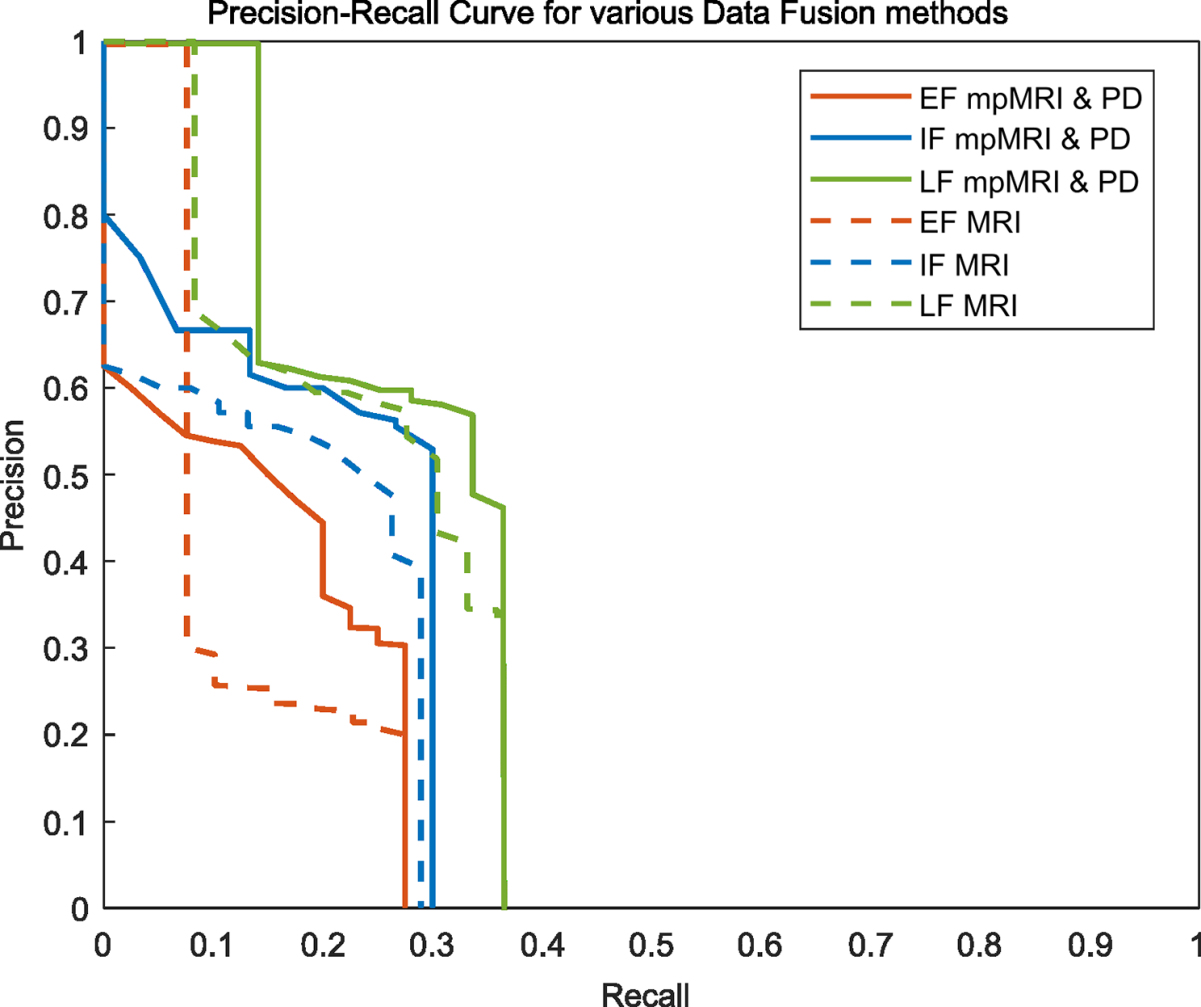
Precision-Recall Curve for various data fusion methods EF (orange), IF (blue), and LF (green) for networks trained with mpMRI and parametric clinical data(solid lines) and mpMRI data only(dotted lines)

**Fig. 4:**
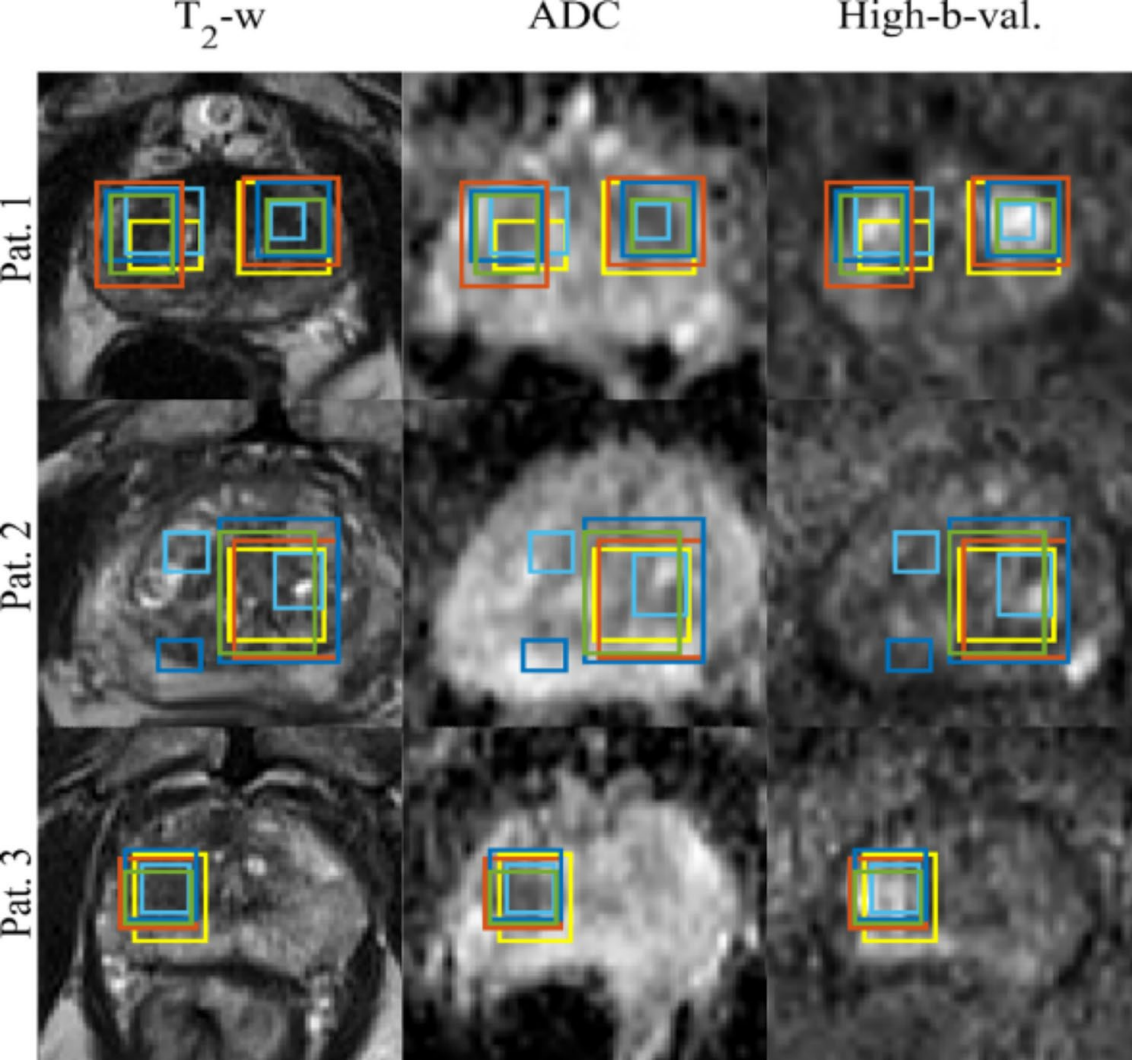
Detected lesions for three patients from the test set by EF fusion network (orange), IF (blue), and & LF (green), ground truth (yellow) and RAD-AI (cyan)

Figure 2 illustrates the overlap between the detected lesions and the ground truth in the test set, using input level, feature level, and decision level fusion strategies. The CNNs achieved a precision of 0.41, 0.51, 0.61 for input level, feature level, and decision level fusion, respectively, while the recall values were 0.18, 0.22, 0.25. Additionally, the AP was 0.13, 0.19, 0.27, and the F scores were 0.55, 0.67, 0.76, respectively for the fusion of image and clinical data. For networks trained only with image data, the CNNs achieved a precision of 0.36, 0.45, 0.56 for input level, feature level, and decision level fusion, respectively, while the recall values were 0.13, 0.21, 0.23. Additionally, the AP was 0.12, 0.16, 0.23, and the F scores were 0.19, 0.26, 0.33, respectively. The network’s performance was further summarized using an area under the precision-recall curve (AUC-PR) in Fig. 3. A two-sided student’s t-test did not reveal any significant differences between the predicted scores of any two networks (*p* = 0.26, 0.62, and 0.85). In Fig. 4, the ground truth and the predicted lesion maps are displayed overlaid along with the ground truth on the corresponding input image data for three test patients from the test cohort. We show test cases with multiple lesions as well as single lesions. In patients 1 and 3 all networks detected multiple and single lesions, while in patient 2 the IF network and the RAD-AI detected a suspected lesion additionally in contrast to the ground truth. Table 3 provides an overview of the comparison of mean DSC obtained by comparing the ground truth with predicted segmentation maps for networks trained with T2w, ADC, and High b-value images only, only with mpMRI data, mpMRI and parametric clinical data and RadAI.

**Table 2:** Overview of the number of patients with various Gleason grades from the two patient cohorts

Gleason Grades	6	7a	7b	8	9
**Irradiation & Prostatectomy (n**_**irr+prost**_)	16	35	38	20	5
**Whole mount Histology (n**_**prost**_)	2	6	7	4	3

## Discussion

In this study, we investigated multiple deep learning data fusion methods for the automatic detection and segmentation of csPCa lesions using a patch-based 3D UNet. The late fusion network performed better with a mean DSC of 0.36 ± 0.24 when compared to the input and feature level fusion networks 0.30 ± 0.23 & 0.34 ± 0.26 respectively. A statistical t-test showed no significant differences in the prediction (*p* = 0.51, 0.10, and 0.82). The EF network offers advantages in cost effectiveness and memory efficiency; however, this approach does not exploit the relationships between the different modalities. Rather, as it learns a joint feature representation, it simply fuses the data at the input level. The IF networks instead capture the complex relationships between the input modalities, by learning both individual and joint feature representations. However, both the EF and IF networks are not flexible to train in case of missing data. Moreover, the LF network only learns an independent feature representation, and is flexible to train such network when one or more input data modality is missing, it fails to learn the joint feature representation, since each network is trained with a particular input data. Though the IF and LF have a better performance; their design leads to an increased number of network parameters, which in turn increase memory consumption and training time, making it computationally expensive to train such networks [17, 47, 48].

In this work we evaluated the influence of combining parametric clinical data with mpMRI images, by comparing the predictions of the networks trained solely on mpMRI data with networks trained on both mpMRI and parametric clinical. By performing a t-test (*p*-value = 0.47, 0.88, 0.28), we found no significant difference in the overall prediction for csPCa detection and segmentation. Multimodal imaging alone could be adequate for training CNNs for csPCa detection and segmentation. However, the inclusion of clinical data could be beneficial for csPCa lesion risk classification.

To determine the importance of each mpMRI sequence in the tasks of lesion detection, we relied on predictions from the independently trained CNNs using T2W, ADC and High-b Value images as inputs (Table 3). For the prostate gland detection, the UNet trained with only T2W images performed the best, in comparison to the networks trained using ADC images or High b-Value images only. This is likely a result of the well-defined prostate anatomy in the T2W sequence thus validating the use of T2W images in for prostate gland & prostate zone segmentation [49–52]. For prostate lesion detection and segmentation, the networks trained with only the ADC and High b-Value images showed an 18% improvement in detection csPCa lesions on the test set, in comparison with the UNet trained with only T2W images. We also showed that the network trained with only T2W images performed better in detecting csPCa lesions with PI-RADS score greater than 4 and 5, a DSC of 0.50, 0.49 & 0.32 and underperformed in detecting csPCa lesions with PI-RADS score 1 to 3, a DSC of 0.17, 0.09, 0.13,0.0 respectively. We compared the AI-Rad generated segmentation mask with PCa-Histo and PCa-CNN (Early-Fusion) Table 3, indicate that our network performed similarly to RAD-AI [43–45] in segmentation of csPCa lesions.

**Table 3:** Mean DSC between the ground truth (csPCa-Histo) segmentation and the predicted segmentation (csPCa-CNN and csPCa-AI-Rad) for various networks

Network	Input Data	Fusion	Mean DSC
UNet	T2W only		0.14 ± 14
	ADC only		0.25 ± 20
	High b-Value only		0.28 ± 21
	mpMRI & parametric clinical data	EF	0.30 ± 0.23
		IF	0.34 ± 0.26
		LF	0.36 ± 0.24
	mpMRI only	EF	0.26 ± 0.22
		IF	0.33 ± 0.23
		LF	0.34 ± 0.26
RADAI	mpMRI only	EF	0.31 ± 0.21

## Conclusion

In this study, mpMRI and the corresponding clinical data were combined to compare the various data fusion methods for CNN-based csPCa detection, segmentation and risk prediction. We evaluated the significance of including clinical data and found no significant improvement in the predictions of the CNNs for Detection and segmentation. The importance of each mpMRI sequence was analyzed and the results illustrated that all the sequences play a critical role in detection and segmentation of csPCa. Combining parametric clinical data with mpMRI data improves risk prediction. Finally, we compared the performance of our network with RAD-AI [43, 45] and found that our network performs comparably to the DI2IN method [44].

## Data Availability

No datasets were generated or analysed during the current study.

## Abbreviations

csPCa: Clinically significant Prostate carcinoma
DRE: Digital Rectal Examination
PSA: Prostate Specific Antigen
PI-RADS: Prostate Imaging Reporting and Data System
MRI: Magnetic Resonance Imaging
mpMRI: multiparametric MRI
ADC: Apparent Diffusion Coefficient maps
PSAD: PSA density
GTV: Gross Tumor Volume
CT: Computed Tomography
PET: Positron Emission Tomography
CNN: Convolutional Neural Network
MLP: Multi-Layer perceptron
EF: Early Fusion
IF: Intermediate Fusion
LF: Late Fusion
AP: Average Precision
DSC: Dice Similarity Coefficient
